# Effects of Single Low-Carbohydrate, High-Fat Meal Consumption on Postprandial Lipemia and Markers of Endothelial Dysfunction: A Systematic Review of Current Evidence

**DOI:** 10.1093/nutrit/nuae103

**Published:** 2024-08-02

**Authors:** Megan L Wilson, Katie E Lane, Abdulmannan Fadel, Ellen A Dawson, Ella Moore, Mohsen Mazidi, Richard J Webb, Ian G Davies

**Affiliations:** Research Institute of Sport and Exercise Sciences, Faculty of Science, Liverpool John Moores University, Liverpool L3 3AF, United Kingdom; Research Institute of Sport and Exercise Sciences, Faculty of Science, Liverpool John Moores University, Liverpool L3 3AF, United Kingdom; Department of Nutrition and Health, College of Medicine and Health Sciences, United Arab Emirates University, Al Ain, United Arab Emirates; Research Institute of Sport and Exercise Sciences, Faculty of Science, Liverpool John Moores University, Liverpool L3 3AF, United Kingdom; Research Institute of Sport and Exercise Sciences, Faculty of Science, Liverpool John Moores University, Liverpool L3 3AF, United Kingdom; Clinical Trial Service Unit, Nuffield Department of Population Health, University of Oxford, Oxford OX3 7LF, United Kingdom; Nutrition and Food Science, School of Health and Sport Sciences, Liverpool Hope University, Liverpool L16 9JD, United Kingdom; Research Institute of Sport and Exercise Sciences, Faculty of Science, Liverpool John Moores University, Liverpool L3 3AF, United Kingdom

**Keywords:** postprandial period, lipemia, dyslipidemia, lipoproteins, lipid metabolism, glycemic control, endothelial dysfunction, cardiovascular disease, ASCVD

## Abstract

**Context:**

Postprandial lipemia (PPL) is associated with increased risk of endothelial dysfunction (ED), a precursor of atherosclerotic cardiovascular disease (ASCVD). The effects of low-carbohydrate, high-fat (LCHF) diets on ASCVD risk are uncertain; therefore, gaining a greater understanding of LCHF meals on PPL may provide valuable insights.

**Objective:**

The current systematic review investigated the effects of single LCHF meal consumption on PPL and markers of ED.

**Data Sources:**

CINAHL Plus, PubMed, Web of Science, and Cochrane Central Register of Controlled Trials (CENTRAL) were searched for key terms related to endothelial function, cardiovascular disease, glycemia, lipemia, and the postprandial state with no restriction on date.

**Data Extraction:**

Full-text articles were independently screened by 2 reviewers, of which 16 studies were eligible to be included in the current review. All trials reported a minimum analysis of postprandial triglycerides (PPTG) following consumption of an LCHF meal (<26% of energy as carbohydrate). Results were reported according to the Preferred Reporting Items for Systematic Reviews and Meta-Analyses (PRISMA) statement.

**Data Analysis:**

Single-meal macronutrient composition was found to play a key role in determining postprandial lipid and lipoprotein responses up to 8 hours post-meal. Consumption of LCHF meals increased PPTG and may contribute to ED via reduced flow-mediated dilation and increased oxidative stress; however, energy and macronutrient composition varied considerably between studies.

**Conclusion:**

Consumption of an LCHF meal had a negative impact on PPL based on some, but not all, single-meal studies; therefore, the contribution of LCHF meals to cardiometabolic health outcomes remains unclear. Further research is needed on specific categories of LCHF diets to establish a causal relationship between postprandial modulation of lipids/lipoproteins and impaired vascular endothelial function.

**Systematic Review Registration:**

PROSPERO registration no. CRD 42023398774.

## INTRODUCTION

Cardiovascular disease (CVD) remains the leading cause of morbidity and premature death worldwide.^1^ A major contributor to the global burden of disease, CVD accounts for more deaths annually than any other noncommunicable disease,^2^ with an estimated 19.05 million deaths in 2020.^3^ The development of CVD is associated with obesity, hypertension, insulin resistance, dyslipidemia, and hyperglycemia, all of which increase CVD risk both independently and as interlinking components of the metabolic syndrome (MetS).^4^^,^^5^ Attributed to underlying metabolic stressors, including dyslipidemia, endothelial dysfunction, and insulin resistance, a 2020 meta-analysis of epidemiological data highlighted the growing prevalence of MetS, which is estimated to affect 12.5%–31.4% of the global population.^6^ While damaging to health independently, the co-presence of 3 or more of the included cardiometabolic abnormalities is an established risk factor for the development of atherosclerotic CVD (ACVD), with individuals diagnosed with MetS twice as likely to develop the disease.^7^^,^^8^

A precursor of cardiovascular events, endothelial dysfunction is considered an early marker for atherosclerotic disease; therefore, maintenance of vascular endothelial homeostasis is of key importance in attenuating ACVD risk.^9^ The pathogenesis of ACVD is multifactorial in etiology and is attributed primarily to the retention and oxidation of apoB-containing lipoproteins, including low-density lipoproteins (LDLs) within the subendothelial intima of the arteries, and increased vascular production of reactive oxygen species (ROS), resulting in a reduction in vasoprotective endothelium-derived nitric oxide (NO) activity and impaired endothelium-dependent vasodilation or endothelial dysfunction.^10–13^ Associations between lipid metabolism and endothelial dysfunction are well established,^14–16^ with physiological changes such as hypertriglyceridemia, small LDL particle size, and low high-density lipoprotein cholesterol (HDL-C) being shown to potentiate impaired endothelial function.^17–19^ A predictive marker of aggregation and atherogenicity irrespective of LDL cholesterol (LDL-C) concentration,^20^ LDL particle size has been shown to play a key determining factor for the development of endothelial injury and subsequent pathogenesis of atherosclerosis.^21^ Several studies have reported associations between small, dense LDL (sdLDL) particles and endothelium-dependent vasodilation,^22^^,^^23^ with elevated concentrations of sdLDL particles found to positively correlate with cardiovascular events. While the exact mechanisms mediating increased atherogenicity of sdLDL particles in comparison to other phenotypes of LDL subclasses are less established, possible underlying mechanisms include increased oxidation, increased binding of sdLDL to the arterial wall, and decreased binding to LDL receptors.^24^

The effects of diet on endothelial function are well known, with studies reporting adverse associations between endothelial function and postprandial hyperglycemia and hyperlipidemia.^25^^,^^26^ For the most part, individuals in post-industrialized societies spend the majority of nonsleeping hours in the postprandial state.^27^ Postprandial hyperglycemia has been shown to attenuate endothelial function by increasing oxidative stress via postprandial glucose (PPG) excursions, thereby impairing flow-mediated dilation (FMD), a noninvasive measure of NO-mediated endothelial function.^28^ In addition, postprandial lipemia (PPL) is also associated with a reduction in FMD, the cause of which has been attributed to a postprandial decrease in NO and increased oxidative stress.^29^ Other measures of endothelial dysfunction include pulse wave velocity (PWV), peripheral arterial tonometry (PAT), and digital pulse wave analysis to assess arterial stiffness and vascular responsiveness.^30^^,^^31^ As such, multiple studies have investigated the cardioprotective effects of diet on endothelial health, with varying results. Diets rich in saturated fatty acids (SFAs) were shown to have a deleterious effect, while diets high in monounsaturated fatty acids (MUFAs) and polyunsaturated fatty acids (PUFAs), such as those found in the Mediterranean diet, have been linked to improvements in endothelial function.^32^ Studies also reported improved PPG excursions following dietary carbohydrate (CHO) restriction in comparison to high-CHO diets, resulting in a reduction in oxidative stress.^33^

Earlier studies, such as those by Sharman et al^34^ and Volek et al,^35^^,^^36^ have highlighted the effects of very-low-CHO diets (VLCDs) on lipoproteins and associated risk factors for CVD. However, while several studies have investigated the postprandial effects of high-fat meals (HFMs) on cardiometabolic risk, no systematic reviews have focused on postprandial metabolic health with respect to low-CHO meals in recent years. Therefore, the aim of the current systematic review was to determine the extent to which meals low in CHO but high in fat, irrespective of fat source, influence cardiometabolic risk markers in the postprandial state in normal healthy adults and those with cardiometabolic risk based on available randomized controlled trials (RCTs) and clinical trials. This review also seeks to contribute to the discussion of the importance of postprandial metabolic status as an indicator of cardiometabolic health, which, in recent years, has become more widely recognized as an independent risk factor for ACVD. The review protocol is listed in the International Prospective Register of Systematic Reviews (PROSPERO; CRD 42023398774).

## METHODS

### Literature search strategy

The current study is a systematic review on the effects of low-CHO, high-fat (LCHF) meal consumption on postprandial markers of cardiometabolic health, with a specific focus on the extent to which low-CHO meals high in MUFAs, PUFAs, or SFAs impact PPL, an independent risk factor for endothelial dysfunction and CVD risk. This review was performed and reported according to the Preferred Reporting Items for Systematic Reviews and Meta-Analyses (PRISMA) statement, and PICOS (Population, Intervention, Comparison, and Outcomes) criteria^37^^,^^38^ were used to define the following research question: “To what extent do meals low in carbohydrates but high in monounsaturated, polyunsaturated, or saturated FAs contribute to PPL response?” (Table 1).

**Table 1.** PICOS Criteria for Inclusion of Studies

Parameter	Inclusion/exclusion criteria
Participants	Adults aged ≥18 y
Interventions	Diet interventions
Comparisons	Placebo or control group, different diet/intake
Outcomes	Primary outcomes included presence of both analysis of endothelial function and postprandial plasma analysis—namely, plasma glucose, lipids, and lipoproteins
Study design	Randomized controlled or clinical trials with either parallel or crossover design

### Search methods

CINAHL Plus, PubMed, Cochrane Central, and Web of Science were searched for relevant randomized trials with no restriction on date or language. Databases were searched individually with advanced search strategies utilizing various combinations of controlled phrases as either keywords or MeSH (Medical Subject Heading) terms. To maximize search sensitivity, multiple terms were combined relating to endothelial function, CVD, the postprandial state, glycemia, and lipidemia to enhance precision (Supplementary Material). The wild-card term “*” was included to increase the sensitivity of the search strategy. Following the initial database search and screening, full-text articles were independently reviewed by 2 of the authors (M.L.W. and E.M.) to ensure all studies included were relevant and met the inclusion criteria for the current study. Furthermore, to minimize the effect of publication bias, a snowball method, characterized by manual checking of references from retrieved articles, was applied to relevant studies that met the selection criteria outlined below.

### Selection criteria

The current review included RCTs and randomized trials that evaluated the effect of LCHF meals (<26% of total energy intake from CHO)^39^ on postprandial lipidemia, glycemia, and/or endothelial function as a primary or secondary outcome. Eligible studies were screened to meet the following criteria: (1) adult study population (aged 18+ years), (2) randomized controlled or uncontrolled clinical trials with either parallel or crossover design, (3) presence of postprandial plasma lipid analysis, and (4) presentation of sufficient information on the primary objective/outcome at baseline and endpoint or provision of net change values. Exclusion criteria were as follows: (1) nonclinical studies, (2) animal and/or in vitro study models, (3) pregnancy/child studies, (4) studies that did not provide baseline or endpoint values for outcomes of interest, and (5) studies where meals were given with supplements or drugs without a control group receiving only an HFM; (6) narrative reviews, opinion pieces, editorials, protocols, and/or studies that did not include primary data were also excluded from the review.

### Data extraction and critical appraisal

**Figure 1.**
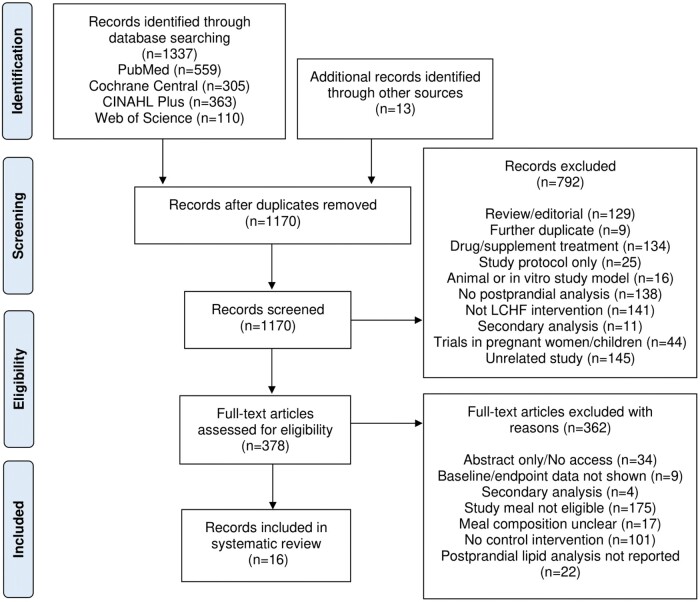
PRISMA Flowchart Illustrating the Screening and Selection Process. *Abbreviations:* LCHF, low-carbohydrate, high-fat; PRISMA, Preferred Reporting Items for Systematic Reviews and Meta-Analyses

Duplicate studies were removed, and the remaining studies were screened by title and abstract initially before full-text articles were reviewed by 2 researchers independently (M.L.W. and E.M.) to minimize risk of bias. Following the initial screening, studies were listed as either included, excluded, or pending if the eligibility of the study to be included in the review was unclear. Pending studies were temporarily included in the next stage of screening. Once retrieved, full-text articles were independently reviewed, and inclusion/exclusion criteria applied. If there was disagreement, a third author (I.G.D.) was consulted to resolve any inconsistencies and reach a consensus. Based on the PRISMA guidance, a flowchart was produced to enable transparency of the screening process (Figure 1).

### Data extraction and management

Full-text studies that met the inclusion criteria were retrieved and screened to assess eligibility by 2 reviewers (M.L.W. and E.M.). Once methodological quality was determined, the reviewer (M.L.W.) extracted and transferred the data to a Microsoft Excel spreadsheet (Microsoft Corporation, Redmond, WA, USA) and briefly summarized the key concepts, findings, and results from each study. Summaries for each study were discussed with a third reviewer (I.G.D) and any inconsistencies resolved. Data were organized by first author, year of publication, country of origin, age range and sex of participants, study design and duration, intervention type, presence of background disease/conditions, and summary of key findings (Table 2).^40–55^

**Table 2.** Characteristics of Included Studies

First author (year), country	Study design	Age range, y	Male, %	Background disease	Sample size, *n*	Study duration	Intervention type	Summary of key findings
Averill et al (2020), USA^40^	Crossover RCT comparing the effects of HCM (500 kcal, 10% fat, 10% protein, 80% CHO) and HSFM (500 kcal, 80% SFA, 10% protein, 10% CHO) meals on postprandial lipids (0–6 h)	18-50	40	Healthy subjects with no history of chronic disease	15	2 test days separated by a washout period of ≥1 wk	2-arm diet intervention: standardized HCM (control) vs HSFM test	No changes in plasma lipids were observed following an HCM. Postprandial TG levels following consumption of an HSFM increased significantly at 3 h in comparison to both baseline and a HCM meal (*P *=* *.0008 and *P *<* *.05, respectively); however, no other between-meal differences were observed at any time point. Following an HSFM, HDL-C levels decreased significantly at 3 h in comparison to baseline (*P *=* *.009). HDL protein (total mass) decreased significantly between baseline and 6 h after an HSFM (*P *=* *.006). HDL TG content also increased by 25% at 6 h postprandially following a HSFM (vs baseline, *P *=* *.02). Apo-A1 significantly increased following consumption of an HSFM (vs baseline, *P *=* *.001). At 6 h, the differences in TG and HDL-C levels were attenuated and not significantly different from baseline. Neither test meal altered levels of TC, LDL-C, or apoB. HSFM induced significant enrichment of HDL in multiple phospholipid species at both 3 and 6-h time points (all *P *<* *.05).
Emerson et al (2017), USA^41^	Crossover RCT comparing the effects of an HFM (1319 ± 338 kcal, 64% fat, 20% protein, 16% CHO), MFM (660 ± 169 kcal, 30% fat, 15% protein, 55% CHO), and BPM (MFM consumed twice, 3 h apart) (0–6 h)	18-35	100	Healthy subjects with no history of chronic disease	9	3 test days separated by a washout period of ≥1 wk	3-arm diet intervention: HFM, MFM, and BPM	A significantly higher TG peak was observed after the HFM than the MFM (*P*= .003), but there were no significant differences between the BPM and the HFM or MFM. Time-to-peak TG response was significantly longer in the HFM (*P*= .01) and BPM (*P*= .01) trials than in the MFM trial, with no difference between the HFM and BPM. The tAUC response for TGs was significantly larger in the HFM trial than in the BPM (*P*= .03) and MFM (*P*= .0005) trials, but there was no significant difference between the BPM and MFM trials. TG iAUC was greater in the HFM trial than in the BPM (*P*= .01) and MFM (*P*= .001) trials, whereas there was no significant difference between the BPM and the MFM trials. Peak LDL-C response was significantly greater in the MFM trial than in the HFM and BPM trials (*P*=.007 for both). The MFM elicited a significantly greater HDL-C (tAUC) response than the HFM (*P*= .02) and the BPM (*P*= .047), but there was no significant difference between the HFM and BPM.
Haddad et al (2014), USA^42^	Crossover RCT comparing the effects of walnut consumption (631 kcal, 83.7% fat, 8.6% protein, 7.8% CHO) or control (621 kcal, 84.3% fat, 8.5% protein, 7.2% CHO) on postprandial oxidative stress (0–5 h)	23-44	37.5	Healthy subjects with no history of chronic disease	16	2 test days separated by a washout period of ≥1 wk	2-arm diet intervention: walnut or control meal	As expected, TG concentrations increased following consumption of both test meals; however, TG (AUC^0-5h^) was significantly higher following the walnut meal in comparison to control (vs baseline, *P *=* *.037). Neither the walnut nor control meal influenced postprandial total cholesterol response expressed as AUC_0-5h_.
Holmer-Jensen et al (2012), Denmark^43^	Crossover RCT comparing the acute effects of milk-derived dietary proteins on PPL as part of an HFM (66% fat, 19% protein, 15% CHO for all meals) (0–8 h)	44-74	45.5	Healthy subjects with no history of chronic disease	11	4 test days separated by a washout period of 2 wk.	4-arm diet intervention: alpha-lactalbumin (ALPHM; 1191 kcal), whey isolate (WI; 1188 kcal), caseino-glycomaceopeptide (CGMPM; 1190 kcal), or whey hydrolysate (WH; 1189 kcal)	No significant differences in plasma TG concentrations or TG fractions were observed between meals.
Khor et al (2014), Australia^44^	Crossover RCT comparing the effects of ice cream (940 kcal, 41.6% fat, 49.5% CHO [as sugar], 8.9% protein), cream (409 kcal, 79.9% fat, 3.8% CHO, 16.6% protein), sugar (388 kcal, 100% CHO), and avocado (940 kcal, 95.2% fat, <1% CHO, 3.8% protein) on postprandial oxidative stress (0–4 h)	21-78	45.5	Healthy subjects with no history of chronic disease	11	4 test days separated by a washout period of 2 wk	4-arm diet intervention: ice cream, cream, sugar, or avocado	No significant differences in plasma TG or cholesterol were observed between time points after ingestion of the ice cream, cream (fat/protein), sugar, and avocado test meals.
Lane-Cordova et al (2016), USA^45^	Crossover RCT comparing the effects of ingestion of a beverage high in SFA or TFA (520 kcal, 95.5% fat, 3.8% CHO, 0.75% protein for both meals) on postprandial vascular endothelial function (0–4 h)	21-65	82	Healthy subjects with no history of chronic disease	11	2 test days separated by a washout period of ≥1 wk (males) and ≥1 mo (females)	2-arm diet intervention: standardized test beverage high in SFAs (control) or TFAs	PPG was significantly reduced across groups (*P *<* *.001) but did not differ significantly between beverages (0–4 h). There was a significant interaction effect between beverage types for insulin (*P *=* *.01), with a trend reported for reduction in insulin after consumption of a high-SFA beverage but not a high-TFA beverage (0–4 h). Serum FFA concentrations significantly increased following both beverages (*P *=* *.03); however, changes in PPTG were nonsignificant for both beverages). FMD (mm Δ) was significantly reduced following consumption of both beverages (*P *<* *.01 for time effect, *P *=* *.034 for interaction effect), with a significant decrease observed following the TFA beverage (*P *<* *.01) and in comparison, to SFA (0–3 h for all, *P *=* *.49). Relative FMD, expressed as %Δ from baseline, also significantly decreased between baseline and post-consumption for both beverages (0–3 h, *P *=* *.014 for main effect).
Lin et al (2020), Canada^46^	Crossover RCT comparing the effects of acute whole-apple consumption on PPL as part of an HFM (control, 729.7–1524.1 kcal; apples, 906–1638.1 kcal; both, 1 g fat/kg body weight, ∼80%-90% of total energy) (0–6 h)	18-70	35	Healthy overweight/obese	26	2 test days separated by a washout period of ≥1 wk	2-arm diet intervention: OFTT (control) consisting of 500 mL 35% fat whipping cream and skimmed milk, standardized with additional skimmed milk powder or OFTT+ 200 g Gala apples)	PPTG concentrations increased significantly in the first 3 h (*P *<* *.05) after consumption of both meals, decreasing after 5 h. Acute apple consumption as part of an OFTT had no effect on PPTG, apoB-48, and time to peak concentration (*T*_max_) compared with an OFTT meal alone. PPG fluctuated over time but there was no significant difference between treatments. In comparison to the OFTT meal, significantly elevated insulin concentrations were observed from 20 to 180 min when combined with apple ingestion (*P *<* *.05).
Lopez et al (2011), Spain^47^	Crossover RCT comparing the effects of meals high in MUFAs or SFAs (both ∼800 kcal, 72% fat, 22% CHO, and 6% protein) on lipid profile and insulin secretion in individuals with elevated fasting TG concentrations (0–8 h)	25-40	100	Healthy subjects with fasting hypertriglyceridemia	14	3 test days separated by a washout period of ∼1 wk	3-arm diet intervention: plain pasta (30 g/m^2^ body surface area), 1 slice of brown bread, and 1 skim yogurt with either olive oil (14.9% SFAs, 81.0% MUFAs, 4.1% PUFAs), butter (65.3% SFAs, 31.3% MUFAs, 3.4% PUFAs), or control (a meal of the same foods with no added fat)	Both HFMs increased mean TG concentrations (*P *<* *.05), peaking at 120 min; however, there were no significant differences between HFMs. All meals induced a decrease in mean plasma NEFA concentrations at 120 min (*P*< .05).
Maki et al (2007), USA^48^	Crossover RCT comparing the effects of oat and bran cereals on PPG and PPL response as part of an HFM (1240 kcal, 76% fat, 16% CHO, 8% protein) (0–10 h)	24-54	100	Healthy subjects with no history of chronic disease	27	2 test days separated by a 2-wk washout and 2-wk intervention period	2-arm diet intervention: oat products (76 g/d of oat bran cereal plus 60 g/d oatmeal) or 81 g/d wheat cereal plus 60 g/d hot rolled wheat cereal (control)	Mean peak TG concentration was significantly lower after oat vs wheat consumption (*P *=* *.016). No statistically significant differences were noted for iAUC TG. No significant differences were observed between oat and wheat meals at any timepoint for NMR lipoprotein subfractions.
Markey et al (2011), Ireland^49^	Crossover RCT comparing the effects of 3 g cinnamon supplementation when combined with an HFM (632 kcal, 65% fat, 12% protein, 23% CHO) on PPL, oxidative stress, and arterial stiffness (0–4 h)	20-30	33	Healthy subjects with no history of chronic disease	9	2 test days separated by a washout period of 4 wk	2-arm diet intervention: consumption of a standardized HFM with 3 g of cinnamon or wheat flour (placebo control)	No significant differences in plasma TC, HDL-C, or LDL-C levels between trials. No significant difference in PPTG between trials; however, there was a main effect for time (pooled placebo and cinnamon data, *P *<* *.05). No significant mean differences between or within groups for arterial stiffness.
McAllister et al (2020), USA^50^	Crossover RCT comparing the effects of varying amounts of MCT oil ingestion (0 g, 28 g, 42 g) on PPL response and oxidative stress (0–4 h)	20-27	100	Healthy subjects with no history of chronic disease	10	3 test days	3-arm diet intervention: ingestion of coffee supplemented with 0 g (control), 28 g, or 42 g of 75% MCT oil and 25% coconut oil	No significant difference between treatments for TG (AUC); however, there was a significant effect for time (*P *=* *.0003). Ingestion of 42 g of MCT resulted in significantly higher TC AUC (*P *=* *.02 and *P *=* *.03) and HDL-C levels (*P *=* *.004 and *P *=* *.037) in comparison to the 0-g and 28-g beverages, respectively.
Pacheco et al (2008), Spain^51^	Crossover RCT comparing the effects of ROO and HPSO as part of an HFM (885 kcal, 72% fat, 22% CHO, 6% protein) (0–8 h)	21-38	100	Healthy and hypertriglyceridemic subjects	28	2 test days separated by a washout period of 1 wk	2-arm diet intervention: consumption of a standardized HFM containing ROO or HPSO	PPTG (netAUC_0–8 h_) was significantly reduced in healthy subjects after the HPSO meal (*P *=* *.046) but did not differ between groups. Postprandial levels of sICAM-1 and sVCAM-1 were significantly reduced following consumption of the ROO meal in both healthy subjects and in normotensive and hypertensive subjects with fasting hypertriglyceridemia (*P *<* *.001, netAUC_5–8 h_).
Rakvaag et al (2019), Denmark^52^	Parallel RCT comparing the effects of supplementation of whey protein, maltodextrin and dietary fiber on postprandial lipid profiles when combined with an HFM (1125 kcal and 55% fat for all meals, WP-LoFi and WP-HiFi meals provided 22% protein and 23% CHO; MD-LoFi and MD-HiFi meals provided 11% protein and 34% CHO) (0–6 h)	55-70	48	Healthy subjects with abdominal obesity	65	2 test days separated by a 12-wk intervention period	4-arm diet intervention: whey protein, low-fiber (WP-LoFi, *n *=* *15); whey protein, high-fiber (WP-HiFi, *n *=* *17); maltodextrin, low-fiber (MD-LoFi, *n *=* *16); and maltodextrin, high-fiber (MD-HiFi, *n *=* *17)	The study found no differences between intervention groups for PPTG (iAUC), FFA, or apoB-100 (both tAUC) following 12-wk consumption of either WP or MD and dietary fiber. PPTG (tAUC) and TC were significantly reduced after WP-LoFi and WP-HiFi interventions compared with MD diets (both *P *<* *.01).
Ruano et al (2005), Spain^53^	Crossover RCT comparing the effects of the phenolic content of virgin olive oil on endothelial reactivity as part of an HFM (75% fat, 21% CHO, 4% protein) (0–4 h)	53-68	24	Hypercholesterolemic subjects with no history of chronic disease	21	2 test days	2-arm diet intervention: consumption of 60 g of white bread and 40 mL virgin olive oil either low (80 ppm) or high (400 ppm) in phenolic compounds	No statistically significant differences were observed for postprandial plasma TG (AUC) or TG in small TRL and large TRL, following consumption of either test meal. Compared with baseline, there were no significant differences in basal and peak IRH values between meals.
Schell et al (2017), USA^54^	Crossover RCT comparing the effects of dried cranberry supplementation when combined with an HFM (974 kcal, 65% fat, 12% protein, 23% CHO) (0–4 h)	50-65	20	Subjects with T2DM and with abdominal obesity	25	2 test days separated by a washout period of ≥1 wk	2-arm diet intervention: consumption of a standardized HFM (control) or HFM + 40 g dried cranberries	PPG was significantly lower following a high-fat meal including cranberries at 2 h and 4 h in comparison to control (*P *<* *.05). Serum insulin and insulin resistance, as assessed by HOMA-IR, did not differ between interventions. Postprandial lipid profiles (TG, LDL-C, HDL-C, TC, and LDL:HDL ratio) were not statistically different following either meal at any time point. Biomarkers MDA and serum IL-18 were significantly reduced at 4 h (*P *<* *.05) following the cranberry meal vs control.
Schmid et al (2015), Switzerland^55^	Crossover RCT comparing the effects of HFMs (60% fat, 18% protein, 22% CHO) containing full-fat milk and dairy products on postprandial inflammatory and metabolic responses (0–6 h)	25-55	100	Healthy subjects with no history of chronic disease	19	3 test days separated by a washout period of ≥1 wk	3-arm diet intervention: high-fat, nondairy control meal (HFC; 1005 kcal), high-fat dairy meal (HFD; 1000 kcal) with cheese products, and a HFM supplemented with 400 mL of whole-fat milk (HFMM; 1277 kcal)	PPTG increased significantly (*P < *.001) in the 4-h period following ingestion of all test meals (high-fat dairy, high-fat nondairy with milk supplementation, and a high-fat control). Postprandial LDL-C and HDL-C decreased significantly across all meals for the same time period (*P < *.001). A significant decrease for PPTG (iAUC) was reported after ingestion of the high-fat dairy meal in comparison to the high-fat milk meal (*P *=* *.02). Full-fat milk and dairy products had no significant impact on the postprandial inflammatory response compared to an HFM.

*Abbreviations:* ALPHM, alpha-lactalbumin; apoA-1, apolipoprotein A-1; apoB, apolipoprotein B; AUC, area under the curve; BPM, biphasic meal; CGMPM, caseinoglycomaceopeptide; CHO, carbohydrate; FFA, free fatty acids; FMD, flow-mediated dilation; HCM, high-carbohydrate meal; HiFi, high-fiber; HDL-C, high-density-lipoprotein cholesterol; HFC, high-fat, nondairy control meal; HFD, high-fat dairy meal; HFM, high-fat meal; HFMM, high-fat, nondairy meal supplemented with milk; HFM, high-fat meal; HSFM, high-saturated-fat meal; HOMA-IR, Homeostatic Model Assessment of Insulin Resistance; HPSO, high-palmitic sunflower oil; iAUC, incremental area under the curve; IRH, ischemic reactive hyperemia; LDL-C, low-density-lipoprotein cholesterol; LoFi, low-fiber; MCT, medium-chain triglycerides; MD, maltodextrin; MFM, moderate-fat meal; NEFA, nonesterified fatty acids; NMR, nuclear magnetic resonance; OFTT, oral-fat-tolerance test; PPG, postprandial glucose; PPL, postprandial lipemia; ppm, parts per million; PPTG, postprandial triglycerides; RCT, randomized controlled trial; ROO, refined olive oil; sICAM-1, soluble intercellular adhesion molecule 1; SFA, saturated fatty acids; sVCAM-1 soluble vascular cell adhesion molecule 1; T2DM, type 2 diabetes mellitus; TC, total cholesterol; TFA, *trans*-fatty acids; TG, triglycerides; TRL, triglyceride-rich lipoproteins; WI, whey isolate; WH, whey hydrolysate; WP, whey protein.

## RESULTS

### Literature search and study selection process

The study selection process that followed the literature search is summarized in Figure 1. A total of 1350 citations were identified through database searches (*n *=* *1337) and screening of references (*n *=* *13). After initial screening and removal of duplicates, 1170 records remained, of which 792 were screened by abstract and excluded. Following the initial screening, 378 full-text articles were retrieved for review and assessed against the established selection criteria.

Three hundred and sixty-two full-text records did not fulfill the inclusion criteria and, after their removal, 16 studies remained eligible to be included in the current review. The reasons for exclusion of the 362 full-text articles are outlined in Figure 1: 175 trials did not include analysis of LCHF meals; 22 trials did not include plasma analysis in the postprandial period; 34 trials were excluded as full-text access was unavailable (abstract/protocol only); 30 studies were excluded due to lack of clarity regarding the macronutrient content of test meal, reporting of baseline data only, or secondary analysis; and 101 trials were omitted due to no control intervention.

The literature search identified 16 studies (total sample size, *n *=* *317) that were either acute (single test days) or longer-term (≥1 month) trials that investigated the effects of LCHF meals (<26% of total energy as CHO) and included measurements of postprandial lipemia and/or endothelial dysfunction as either a primary or secondary outcome. Some studies also included other postprandial cardiometabolic markers that were included in our PICOS statement. The 16 studies are summarized in Table 2.^40–55^

### Characteristics of the included studies

The characteristics of the included studies are summarized in Table 2.^40–55^ Published between 2005 and 2020, the included studies originated from 7 countries including the United States (7 studies), Spain (3 studies), Denmark (2 studies), and 1 study each from Canada, Australia, Switzerland, and Ireland. The number of participants included in each study ranged from 9 to 65.^41^^,^^49^^,^^52^ Of the 16 studies that were included, all consisted of RCTs, of which 15 utilized a crossover study design.

With respect to interventions that studied the effects of diet to assess the extent to which postprandial lipid responses and endothelial function may be affected, studies varied from 9 days^40^^,^^42^^,^^46^^,^^51^^,^^53^^,^^54^ to 12 weeks^52^ in duration, with participant age ranging from 18 to 70 years. Trials were conducted in both sexes, with the exception of studies by Emerson et al,^41^ Lopez et al,^47^ Maki et al,^48^ McAllister et al,^50^ Pacheco et al,^51^ and Schmid et al,^55^ which were performed only in male cohorts. Test meal composition and energy content varied across studies, from 388 to 1638 kcal per meal,^44^^,^^46^ with macronutrient intake varying from 0% to 23% CHO, 0% to 22% protein, and 55% to 95.5% fat, respectively.^45^^,^^49^^,^^50^^,^^52^^,^^54^ At a minimum, all studies measured postprandial values at baseline (0 minutes) and postintervention following completion of the study (average duration of 4 to 10 hours post-meal). Several biochemical techniques were used to assess the characterization of lipid profiles including nuclear magnetic resonance (NMR) spectrophotometry and enzymatic analysis. Quantification of PPG and lipid markers was also measured enzymatically, whereas endothelial function was assessed using PWV and FMD of the brachial artery.^56^

### Impact of LCHF meal consumption on PPL response in single-meal interventions

Previous literature shows that macronutrient intake can have both adverse and favorable effects on postprandial cardiovascular metabolism.^57^^,^^58^ Of the 16 trials retrieved, all studied the immediate metabolic response after a single HFM, particularly the control of triglyceride (TG) and lipoprotein changes for up to 10 hours post-consumption. Averill et al^40^ investigated the postprandial effects of a single meal (high-CHO meal [HCM] vs high-saturated-fat meal [HSFM]) on TG, HDL-C, and the lipidome and proteome of the HDL particle. The results revealed significantly elevated postprandial triglycerides (PPTG) 3 hours after the HSFM compared with baseline (*P *=* *.0008). In contrast, HDL-C decreased significantly at the same time point and meal (*P *=* *.009). After 6 hours, TG and HDL-C were no longer significantly different from baseline values, indicating clearance of exogenous TG. Advanced targeted analysis of the HDL lipidome and proteome using parallel reaction monitoring liquid chromatography mass spectrometry found significant effects of meal macronutrient composition for HDL relative enrichment in protein, TG, and phospholipid content, but not cholesterol ester or free cholesterol content (all expressed as % of total HDL mass). Consumption of an HSFM resulted in relative depletion of HDL protein and increased total phospholipid content at 6 hours (*P *=* *.006 and *P *=* *.009, respectively). A 25% increase in enrichment of HDL TG was also observed compared with baseline (*P *=* *.02). In contrast, the abundance of HDL protein and TG remained consistent following an HCM. Despite significant between-meal differences in HDL phospholipid % observed at 6 hours (*P *=* *.006), with the exception of PPTG, the study reported no significant differences between meals at any time point for concentrations of total cholesterol (TC), LDL-C, apoA-1, or apoB.

Likewise, a 2017 study by Emerson et al^41^ sought to investigate the effects of realistic test-meal protocols on PPL and inflammatory responses in comparison to a standard HFM. The 3-arm diet intervention compared the effects of an HFM, moderate-fat meal (MFM), and biphasic meal (BPM), at which point the MFM was consumed twice within a 30-hour period. As expected, consumption of an HFM resulted in significant elevations of TG, TG (incremental area under the curve [iAUC]), and greater time-to-peak (*T*_max_) response in comparison to the MFM (*P *=* *.003, *P *=* *.001, and *P *=* *.01, respectively), with no differences observed between the HFM and BPM. Conversely, the study reported significant meal effects for both HDL-C and LDL-C (total area under the curve [tAUC]) responses following the MFM in comparison to the HFM (*P *=* *.02 and *P *=* *.0009, respectively), with no significant meal effects for PPG across trials. Similarly, a study by McAllister et al^50^ examined the fat-dosing effects of acute coffee ingestion supplemented with either 0, 28, or 42 g of lipids in the form of 75% medium-chain TG (MCT) oil and 25% coconut oil on plasma markers of oxidative stress in 11 healthy, physically active men. Despite a significant effect for time (*P *=* *.0003), the trial observed no differences between treatments for TG (AUC). However, ingestion of 42 g of MCT oil was shown to significantly increase TC and HDL-C concentrations in comparison to beverages containing 0 g and 28 g of lipids (TC, *P *=* *.02 and *P *=* *.03; HDL-C, *P *=* *.004 and *P *=* *.037, respectively).

Lopez et al^47^ sought to compare the effects of meals rich in MUFAs or SFAs on lipid concentrations in a sample of 14 individuals with fasting hypertriglyceridemia. The single-meal crossover trial compared the effects of 3 isocaloric meals containing no fat (control) or enriched with either olive oil or butter on postprandial lipid profiles and insulin secretion. Consumption of both HFMs significantly increased plasma TG and TG (iAUC_0-8 h_) concentrations, peaking at 120 minutes (*P *<* *.05). However, consumption of a meal high in MUFAs was shown to attenuate PPL response in comparison to an SFA-rich meal in participants with hypertriglyceridemia (*P *<* *.05). In addition, all meals induced a significant decrease in mean non-esterified fatty acid concentrations at 120 minutes (*P *<* *.05). A similar study by Lane-Cordova et al^45^ also investigated the role of different types of fat, specifically the differential effects of dietary fatty acid types on postprandial response and vascular endothelial function. The single-meal crossover trial compared the effects of 2 isocaloric beverages, 1 high in *trans*-fatty acids (TFAs) and a beverage high in SFAs. Post-intervention, PPG decreased across groups (3–4 hours; *P *<* *.001); however, there was no significant difference between test beverages. In addition, there was a significant interaction effect between beverage types for insulin (3–4 hours; *P *=* *.01), with a trend toward reduction in insulin after consumption of the SFA beverage in comparison to the TFA beverage (3–4 hours; *P *=* *.069 for interaction). As expected, serum free fatty acid (FFA) concentrations increased significantly following both beverages (*P *=* *.03), while PPTG remained unchanged across groups, with no significant differences in circulating lipids between meals.

### Influence of single LCHF meal consumption on markers of endothelial function

In addition to investigating the impact of LCHF meal consumption on PPL, 2 trials studied the effects of different classifications of fatty acids consumed as part of an HFM on markers of postprandial endothelial function.^45^^,^^47^^,^^51^ Lane-Cordova et al^45^ compared the effects of 2 isocaloric beverages, 1 high in TFAs and a beverage high in SFAs, on postprandial vascular endothelial function, measured by changes in FMD. Notably, FMD was significantly reduced following consumption of both beverages (3–4 hours; *P *<* *.01 for time effect, *P *=* *.034 for interaction effect), with a significant decrease in relative FMD (%Δ) also reported between baseline and post-consumption (3–4 hours; *P *=* *.014). However, a decrease in absolute FMD (mmΔ) was significantly greater following the TFA beverage (*P *<* *.01) compared with the SFA beverage, suggesting that a single meal high in TFA, in comparison to SFA, attenuates endothelial function through reduction in FMD.

In contrast, an earlier study by Pacheco et al^51^ evaluated the effects of meals high in refined olive oil (ROO) and high-palmitic sunflower oil (HPSO) on markers of endothelial activation and vascular inflammation in participants with normotensive and hypertensive fasting hypertriglyceridemia. Despite a significant reduction in PPTG (netAUC_0–8 h_) in healthy participants following the HPSO meal (*P *=* *.046), TG did not differ between intervention groups. However, postprandial concentrations of soluble intracellular adhesion molecule 1 (sICAM-1) and vascular cell adhesion molecule 1 (sVCAM-1) were significantly reduced following consumption of the ROO meal in both subgroups (*P *<* *.001, netAUC_5–8 h_), suggesting that consumption of a meal high in ROO may have a beneficial effect on markers of vascular endothelial function and inflammation in the postprandial state.

### The role of antioxidant and phytochemical supplementation on PPL response and oxidative stress

In addition to studies that investigated the effects of an LCHF meal on postprandial lipid response as a primary outcome, several studies utilized a single LCHF or HFM combined with a supplementation intervention, and therefore were also included in the current review. Six studies evaluated the impact of dietary antioxidant and polyphenolic flavonoids combined with a single HFM on postprandial metabolic response and markers of oxidative stress.^42^^,^^44^^,^^46^^,^^49^^,^^53^^,^^54^ Haddad et al^42^ hypothesized that consumption of 90 g of walnuts as part of a single HFM would improve postprandial biomarkers in a sample of 16 healthy participants. As expected, TG concentrations increased from baseline following the consumption of both test meals, reaching significance following the consumption of the walnut meal in comparison to control (AUC^0-5 h^; *P *=* *.037). However, neither meal resulted in significant changes in serum cholesterol. Similarly, a 2014 study by Khor et al^44^ investigated the effects of food components of varying phytonutrient content on postprandial oxidative stress, with marginal results. Despite an increase in plasma TG post-consumption of 3 test meals, including ice cream, cream, and avocado at 4 hours (*P *<* *.001, *P *<* *.01, and *P *<* *.05, respectively), no significant differences in TG or TC were observed between meals at any time points. Likewise, an earlier study by Markey et al^49^ observed a main effect for PPTG over time (*P *<* *.05, 0–4 hours), but no significant changes in TG, TC, HDL-C, LDL-C, or digital volume pulse as a measure of arterial stiffness between treatments when considering the effects of 3 g cinnamon supplementation as part of an HFM on measures of postprandial metabolic response. This is in agreement with the earlier findings of Ruano et al,^53^ which reported no significant differences in PPL response in plasma TG (AUC) or fraction analysis of TG in both small and large TG-rich lipoproteins (TRLs) following consumption of an HFM containing 80 parts per million (ppm) or 400 ppm of polyphenolic compounds in the form of virgin olive oil. Polyphenolic content also had no effect on basal or peak ischemic reactive hyperemia as a marker of endothelial reactivity when compared with baseline. In contrast, biomarkers of lipid peroxidation and oxidative stress were significantly improved 120 minutes post-consumption of the high-phenol HFM, with a notable increase in nitrates (NO_[x]_) and lipoperoxides (LPO) (*P *<* *.001 and *P *<* *.005, respectively), and a reduction in 8-epi prostaglandin-F (8-epi-F_2α_) (*P *<* *.001) in comparison to the meal with a lower phenol content. As such, Ruano et al^53^ concluded that consumption of an HFM high in polyphenols may improve vascular endothelial function by reducing oxidative stress and increasing NO metabolites.

Schell et al evaluated the effects of bioactive dietary compounds in the form of cranberry supplementation as part of an HFM in modulating PPTG, PPG, and inflammation in obese individuals with type 2 diabetes mellitus (T2DM). The single-meal crossover study found that PPG was significantly improved following an HFM including 40 g of commercially available dried cranberries at 2 hours and 4 hours in comparison to the control meal (*P *<* *.05). Biomarkers of lipid peroxidation and inflammation were also significantly reduced, with decreased concentrations of malondialdehyde and serum interleukin (IL)-18 detected at 4 hours following the cranberry meal (*P *<* *.05). However, although supplementation of cranberries as part of an HFM was shown to improve PPG excursions, the study reported no significant differences in postprandial lipid profile, serum insulin, or insulin resistance (assessed by HOMA-IR [Homeostatic Model Assessment of Insulin Resistance]) following either meal. Similarly, a more recent study by Lin et al^46^ examined the effects of whole-apple consumption as part of an HFM on PPTG as a risk factor for CVD in healthy overweight and obese individuals. As expected, PPTG concentrations increased significantly in the first 3 hours (*P *<* *.05) after consumption of both HFMs, returning to non-significance after 5 hours. In contrast, the study found that, despite fluctuations of PPG over time, PPG excursions remained unchanged across meals. However, acute apple consumption was shown to increase insulin concentration, reaching statistical significance between 20 and 180 minutes (*P *<* *.05), in comparison to control. Acute apple consumption also had no effect on PPTG, apoB-48, or time to peak concentration (*T*_max_) in comparison to an HFM alone. The study concluded that the addition of whole apples when consumed as part of an HFM did not influence PPL in otherwise healthy obese and overweight individuals.

### Impact of dairy and high-fiber products on PPL

Although most studies retrieved in the current search focused primarily on the acute impact of diet-induced modulation on postprandial metabolic response, 3 short-term studies (> 4 weeks) highlighted the differential effects of dairy-derived products in modulating PPL.^48^^,^^52^^,^^55^ Schmid et al^55^ sought to investigate the effects of full-fat milk and dairy products on postprandial inflammatory and metabolic responses in healthy men. The 3-arm crossover trial observed significant increases in net iAUC for PPTG (*P < *.001 for all) in the 6-hour period following ingestion of all of the meals (high-fat, nondairy control meal [HFC], high-fat dairy meal [HFD], high-fat, nondairy meal supplemented with milk [HFMM]); however, both LDL-C and HDL-C were considerably reduced across all meals for the same time period (*P *<* *.001). Nevertheless, meal-specific alterations in TG were reported, with a significant decrease in TG (iAUC) noted after ingestion of a high-fat dairy meal containing cheese and butter in comparison to a high-fat milk meal (*P *=* *.02). The study also reported significant alterations in postprandial inflammatory markers, including IL-6, tumor necrosis factor α (TNF-α), and endotoxin across all meals. Serum concentrations of IL-6 increased at 6 hours after ingestion of all of the 3 test meals; however, this increase was only significant after the HFC and HFD meals (*P *<* *.001 and *P *<* *.01, respectively) and did not differ between test meals. Likewise, endotoxin concentrations also increased significantly at 6 hours compared with baseline (*P *<* *.001), but this increase did not differ between interventions. A significant reduction in TNF-α was also observed after the HFD meal in the subgroup of participants with a body mass index (BMI) ≤ 25 kg/m^2^. In this subgroup, while a significant difference between the 3 meals was observed with a greater decrease in TNF-α concentration after the HFD meal in comparison to other test meals (*P *=* *.02), this was not evident in participants with a BMI >25 kg/m^2^. As such, Schmid et al^55^ concluded that consumption of full-fat milk and dairy products had no significant impact on the inflammatory response to an HFM in healthy individuals.

A 2019 study by Rakvaag et al reported similar findings investigating the addition of supplementary whey protein and dietary fiber on the lipid profile in 65 individuals with abdominal obesity. Utilizing a double-blind, parallel design, participants were randomly allocated to 1 of 4 groups in a 2 × 2 factorial design: whey protein (WP) and low fiber (WP-LoFi), WP and high fiber (WP-HiFi), maltodextrin (MD) and low fiber (MD-LoFi), or maltodextrin and high fiber (MD-HiFi) for a duration of 12 weeks. Administration of a high-fat standardized mixed-meal test pre- and post-intervention was used to assess alterations in postprandial metabolism. Following consumption of an HFM containing 70 g of fat, the study found no differences between intervention groups for PPTG (iAUC), FFA, apoB-100 tAUC, HDL-C, or LDL-C following 12-week consumption of either WP or MD and dietary fiber. However, PPTG (tAUC) decreased significantly after WP intake in comparison to MD (*P *<* *.01), suggesting that intake of WP in combination with LoFi cereal products for 12 weeks had a beneficial effect on PPTG tAUC but not iAUC in individuals with abdominal obesity. This in in agreement with the earlier findings of Holmer-Jensen et al,^43^ who also observed no significant differences in PPTG, supernatant TG, or infranatant TG between test meals in a 2011 study investigating the acute differential effects of milk-derived dietary proteins on PPL as part of an HFM. Discordant with the observations of Rakvaag et al,^52^ a 2007 study by Maki et al^48^ reported a significant improvement in peak TG concentrations following consumption of a high-fiber oat cereal in comparison to wheat as part of an HFM (*P *=* *.016). However, the study found no significant differences between meals for TG (iAUC) or lipoprotein subfractions at any time point as determined by NMR.

### Assessment of risk of bias

**Figure 2.**
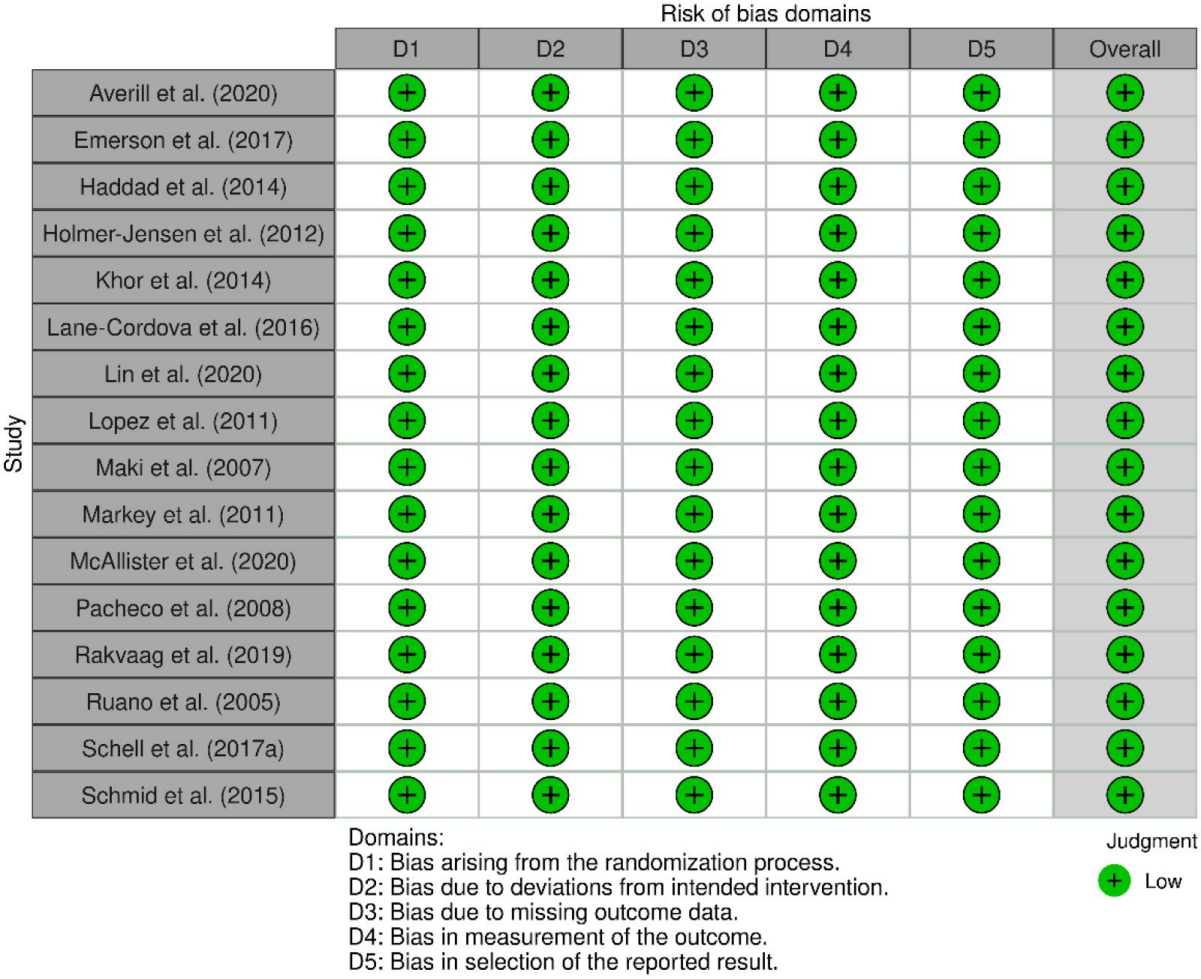
Quality Assessment of Included Randomized Trials (*n *=* *16) Using the Cochrane Risk of Bias Tool^60^

Risks of bias across all included studies were assessed using the Cochrane Risk of Bias Tool.^59^ Due to the nature of several studies, randomization and blinding of participants and/or outcome assessors was not possible; however, as all outcome measures were objective, it was determined that it was unlikely that this influenced the results of the studies. Overall, no study included in the review received a “high” risk of bias result in any assessed category (Figure 2).^40–55^

## DISCUSSION

To our knowledge, this is the first systematic review to comprehensively investigate the impact of LCHF meal consumption on metabolic parameters attributed to PPL and associated microvascular endothelial function in human subjects. Despite heterogeneity across trials, the findings of the 16 included studies, summarized in Table 2,^40–55^ indicate an effect between the consumption of LCHF meals and postprandial lipemia in both healthy adults and individuals with pre-existing metabolic conditions, including T2DM, obesity, hypertension, and hypertriglyceridemia, all of which remain correlating risk factors for ACVD.^61^

The postprandial metabolic response continues to emerge as a leading precursor of cardiometabolic disease risk,^62^ particularly the development of dyslipidemia, which is an independent risk marker of endothelium-derived ACVD.^63^ Given that humans continue to spend a greater proportion of waking hours in a postprandial state, it is imperative that the mechanisms underpinning diet-induced metabolic responses in the post-consumption period are studied further. Consumption of a single HFM is associated with deleterious alterations in postprandial lipoprotein concentrations, specifically elevation of circulating plasma TG.^64^ Several studies have previously investigated the efficacy of very-low-CHO, high-fat diets, characterized by CHO intake comprising 5%–26% of total energy, on CVD risk, with varying results.^65–68^ However very few have studied the effects of single LCHF meal consumption and, more broadly, single-meal macronutrient composition on PPL, lipoprotein subclass distribution, and concomitant endothelial function.

The findings of the current review suggest that consumption of a single LCHF meal did not improve PPL in acute dietary intervention studies. As expected, several studies reported significant transient increases in PPTG following consumption of an LCHF meal, consistent with a high intake of dietary fat.^69^ However, despite significant PPTG time × effect interactions,^40–42^^,^^46^^,^^47^^,^^50^^,^^55^ the majority of included studies failed to observe significant alterations in concentrations of TG, TC, HDL-C, and LDL-C between groups or in comparison to baseline, suggesting that consumption of an LCHF meal neither improved nor adversely affected postprandial lipoprotein concentrations. Given the acute nature of single-meal studies, lack of clinically relevant associations between LCHF meal consumption and changes in PPTG may be attributed to delayed clearance of TRLs, resulting in increased chylomicron and very-low-density lipoprotein (VLDL) remnants, consistent with persistent hypertriglyceridemia. Furthermore, this delayed response, which is associated with pro-atherogenic subendothelial retention of lipoproteins, may also apply to alterations of lipoprotein particle composition. The findings of the current review indicate that, while the anticipated lipemic effect of dietary fat intake was evident at 3–4 hours, structural modulation of lipoprotein particles was not observed until 6 hours postprandially, suggesting that remodeling of lipoproteins, specifically HDL, is deferred in comparison to PPTG response.^40^

Carbohydrate restriction has previously been acknowledged as an effective alternative for the prevention and management of MetS and atherogenic dyslipidemia, with positive improvements reported in PPL, HDL-C, and LDL subclass distribution.^34^^,^^36^^,^^68^ A 2004 study by Volek et al^35^ reported a significant reduction in PPL and preservation of circulating HDL-C following short-term consumption of a VLCD (< 10% of energy from CHO) in comparison to low-fat diet. Similarly, Krauss et al^68^ observed significant reductions in TG and sdLDL, and increased levels of large buoyant LDL particles following consumption of a low-saturated-fat diet with moderate CHO restriction (<26% of energy), substantiating the earlier findings of Sharman et al,^34^ who found that a VLCD improved characteristics of MetS, and improved PPL and LDL subclass distribution in overweight men. It is also important to note that the existing literature predominantly focused on weight loss rather than normocaloric diets, with analysis of lipoproteins performed in a fasted state. Consequently, the hypotriglyceridemic effect of a VLCD may be attributed to body mass reductions and subsequent PPL independent of diet/meal composition.^35^ However, more recent studies have highlighted the potential of LCHF diet adaptation in modulating lipid energy metabolism via increased oxidation of fatty acids and upregulation of lipoprotein lipase (LPL).^70^^,^^71^ Therefore, it may be argued that the consumption of an LCHF diet may improve the lipid profile by increasing expression of LPL, accelerating LPL-mediated lipolysis and turnover of TRLs, resulting in a reduction in VLDL and sdLDL, and enrichment of HDL-C subclasses.^72^

Postprandial TG has been widely reported as a mediator of microvascular endothelial function,^64^^,^^73^ resulting in increased fatty acid oxidation and oxidative stress, and reduced NO bioavailability, thereby impairing endothelial function.^74^ Acute consumption of an HFM is associated with adverse changes in FMD; however, the extent to which LCHF meals affect FMD response remains unquantified. A 2022 meta-analysis of 90 studies by Fewkes et al^75^ determined a transient causal link between single HFM consumption, but not LCHF, and endothelial function, characterized by a decrease in postprandial FMD from 0 to 4 hours post-meal. Identifiable moderators of postprandial FMD are considered to include age, BMI, and meal fat content, the latter of which was inversely associated with adverse changes in FMD (2–4 hours; all *P *<* *.01). Conversely, CHO-restricted diets have been shown to have a negligible effect on endothelial function,^76^ despite favorable improvements in the lipid profile, as evidenced by a recent study by Gram-Kampmann et al,^77^ which concluded that consuming an LCHF diet for 6 months did not affect FMD of the brachial artery in individuals with T2DM.

Of the 16 studies retrieved, only 2 investigated the effects of an LCHF meal and PPL on postprandial endothelial function,^45^^,^^51^ of which only 1 study assessed FMD of the brachial artery as a marker of vasodilatory reactivity.^45^ Lane-Cordova et al^45^ reported a significant reduction in relative FMD (%Δ) between baseline and post-consumption of 2 test beverages high in SFAs and TFAs, respectively (*P *<* *.01 for time effect, *P *=* *.034 for interaction effect). Despite nonsignificant changes in PPTG, a significant reduction in absolute FMD (mmΔ) was reported following both beverages (*P *<* *.05), with a greater decrease following the TFA beverage (*P *<* *.01) compared with SFAs (*P *=* *.49). This is consistent with the earlier findings of de Roos et al,^78^ which reported an immediate deleterious effect on vascular reactivity assessed by a significant reduction in postprandial FMD following a 4-week substitution of TFAs for SFAs as part of an isocaloric high-fat diet, independent of changes in blood pressure and circulating lipids. Given the paucity of studies investigating LCHF meal consumption on FMD, further research is needed to clarify the extent to which single-meal fat percentage and fatty acid composition contribute to postprandial vascular reactivity.

Meal fatty acid composition has been shown to play a key role in the determination of PPL response and endothelial function,^79–81^ with supplementation of PUFAs resulting in significant improvements in both FMD and PWV, a marker of vascular elasticity, in short- and long-term studies.^82–84^ However, evidence is conflicting regarding the influence of single-meal fatty acid composition on PPTG concentrations due to confounding variables resulting from a lack of standardization across studies. To date, few acute studies have evaluated the effects of single-meal fatty acid composition on PPL and FMD as a marker of postprandial endothelial dysfunction.^85^^,^^86^ The findings of the current review suggest that PPL response may be dependent on the source of fat used irrespective of individual metabolic status.^87^ Schmid et al^55^ attributed meals high in SCFAs and medium-chain fatty acids (MCFAs) to a significant improvement in PPTG (iAUC) concentrations post-consumption of a high-fat dairy meal in comparison to a high-fat milk alternative (*P *=* *.02). This was concordant with the more recent findings of Panth et al,^86^ who observed a marked reduction in postprandial hyperlipidemic response following a meal high in MCFAs in comparison to SCFAs and long-chain fatty acids.

With the exception of 3 studies,^45^^,^^47^^,^^51^ none of the included trials compared the effects of fatty acid type, which has been shown to have differential effects on PPL and vascular function in the postprandial state.^88^ Only 2 trials compared the differential effects of different classifications of fatty acids on PPL response and vascular function.^45^^,^^51^ Furthermore, none of the included studies investigated the differential effects of acute CHO restriction combined with a high-PUFA meal on amelioration of vascular function and vasodilation in the postprandial state. Supplementation with omega-3 long-chain fatty acids (LCn-3PUFAs) is associated with favorable alterations of PPTG and FMD in healthy individuals.^89^ Considered cardioprotective at the vascular endothelial level, diets rich in LCn-3PUFAs have been shown to improve postprandial microvascular reactivity and endothelium-independent vasodilation, independently of oxidative stress.^90–92^ As such, the differential effects of fatty acid composition on PPL and vascular reactivity in response to an LCHF meal warrant additional investigation; further research is required to elucidate the extent to which fatty acid composition, in combination with CHO restriction, contributes to the postprandial impairment of endothelial function.

### Strengths and limitations

A strength of the study was the utilization of multiple databases and manual searching of citations, featured in key studies in postprandial metabolism. This approach maximized access to trials that encompassed a broad spectrum of population and methodological considerations, while meeting strict inclusion and exclusion criteria. This allowed for effective identification of comparisons and contrasting associations between studies. In addition, while the study sought not to limit the breadth of trials through exclusion of both healthy individuals and those with chronic disease, it is important to acknowledge that different baseline characteristics, such as previous disease, genetic background, and age, have been shown to affect PPL responses and therefore may not be comparable.^57^^,^^73^^,^^93^

Given the limited number of papers on LCHF single-meal (<26% of total energy) individual postprandial glycemic/lipidemic response and endothelial dysfunction, the inclusion criteria were designed to include all available studies to provide a broad comprehensive review.

The presented study had several limitations. It is important to note that, due to the presence of considerable clinical and methodological heterogeneity across studies, a statistical meta-analysis/meta-regression was not performed. Heterogeneity in the current review was determined by several factors, including the type and composition of test meals used to evaluate PPL response, frequency of time points analyzed, type of intervention, methods of analysis used to study PPL, study duration, and sample size. Sample size and duration varied significantly across studies, all of which were acute interventions (≤16 weeks). Trials ranged from *n* = 5 to 92^87^^,^^94^ participants, with each study consisting of 1–4 postprandial test visits, a factor that may have limited the statistical power of the included studies and potentially compromised the research outcomes.^95^ In addition, due to variations in protocol, specifically the type of nutritional intervention used to elicit the postprandial response, findings from individual postprandial studies were inconsistent, as several studies included postprandial assessment with an LCHF meal as an outcome rather than exposure to the meal itself. Studies included in the current review utilized different types of oral-fat-tolerance tests or fat overloads, ranging from 100% fat preparations, test beverages, and heavy cream to mixed-meal tests with real-life application to evaluate the effects on PPL. Carbohydrate and protein intake also varied considerably across studies from 0% to 23% and 0% to 22% of total energy, respectively,^49^^,^^50^^,^^52^^,^^54^ making it difficult to establish direct causal associations between PPL and LCHF single-meal consumption. Furthermore, comparator meals also varied considerably across studies, particularly with respect to FMD, in which only 1 study compared different types of fat on postprandial vascular endothelial function.^45^ As such, the addition of a standardized test meal protocol for the determination of diet-induced PPL and endothelial dysfunction, along with further comparison of LCHF meals with standardized low- or moderate-fat meals that reflect international dietary recommendations, is needed to provide a strong comparative basis for further analysis.

Moreover, while all studies included postprandial plasma lipoprotein cholesterol analysis, the methods of analysis utilized varied across trials. Although most studies included traditional enzymatic methods as a quantifiable measurement of absolute lipoprotein cholesterol concentrations, few included advanced evaluation of exogenous and endogenous lipoproteins or assessment of post-meal alterations of endothelial function. This is important to note due to the complexity of LDL particles and the propensity of LDL aggregation adversely affecting endothelial cells. Subsequently, advanced analysis of LDL subclasses, including classification of particle size, along with lipid, proteome, and metabolome profiling, may play a key role in ameliorating CVD risk. As such, the findings of the current review confirm a dearth of literature regarding the effect of LCHF diets on both typical and advanced postprandial markers used to quantify lipids and lipoproteins.

## CONCLUSION

We offer valuable insights into cardiometabolic health by examining the effects of consuming a single LCHF meal on PPTG, lipoprotein-lipid levels, and microvascular endothelial dysfunction. The lack of available studies on single-meal macronutrient composition and postprandial markers of cardiometabolic risk remains a concern. As a result, the contribution of LCHF meals to cardiometabolic health outcomes remains unclear. Furthermore, the absence of lesser reported factors, such as quantification of lipoprotein complexity, adds to this uncertainty. To summarize, the consumption of an LCHF meal did not result in a notable improvement in PPL response based on single-meal studies. Consequently, additional research is necessary to investigate the effects of LCHF meals not only on conventional PPL measures but also on the intricate nature of lipoprotein particles in the postprandial state and their potential impact on endothelial dysfunction and subsequent CVD risk.

## Supplementary Material

nuae103_Supplementary_Data

## Data Availability

The complete search strategy can be accessed at https://www.crd.york.ac.uk/PROSPEROFILES/342414_STRATEGY_20220626.pdf.
