# Supplementary material for: Effects of Single Low-Carbohydrate, High-Fat Meal Consumption on Postprandial Lipemia and Markers of Endothelial Dysfunction: A Systematic Review of Current Evidence
Source: Nutr Rev. 2024 Aug 2;83(3):e1049–67. doi: 10.1093/nutrit/nuae103 (PMC11819491; doi:10.1093/nutrit/nuae103)
Supplement: nuae103_Supplementary_Data [file nuae103_supplementary_data.zip › nuae103_Supplementary_Data/342414_STRATEGY_20220626.pdf]

Database	S#	Search Term
CINAHL+ (EBSCO)	#1	Diet, carbohydrate-restrict* [ch] ti. ab. tx.
	#2	Low carb* [ch] ti. ab. tx.
	#3	Diet, ketogenic [ch] ti. ab. tx.
	#4	Atkins ti. ab. tx.
	#5	High fat ti. ab. tx.
	#6	Keto* ti. ab. tx.
	#7	LCHF ti. ab. tx.
	#8	#1 OR #2 OR #3 OR #4 OR #5 OR #6 OR #7
	#9	Endothelium [ch] ti. ab. tx.
	#10	Vascular endothelium ti. ab. tx.
	#11	Endothelial dysfunction ti. ab. tx.
	#12	Inflammation [ch] ti. ab. tx.
	#13	Oxidative stress [ch] ti. ab. tx.
	#14	Atherosclerosis [ch] ti. ab. tx.
	#15	Coronary disease* [ch] ti. ab. tx.
	#16	Cardiovascular disease* [ch] ti. ab. tx.
	#17	Vasoconstriction [ch] ti. ab. tx.
	#18	ASCVD ti. ab. tx.
	#19	#9 OR #10 OR #11 OR #12 OR #13 OR #14 OR #15 OR #16 OR #17 OR #18
	#20	#8 AND #19
	#21	Postprandial ti. ab. tx.
	#22	Post-prandial ti. ab. tx.
	#23	Postprandial period [ch] ti. ab. tx.
	#24	Postmeal ti. ab. tx.
	#25	Post-meal ti. ab. tx.
	#26	Post-eating ti. ab. tx.
	#27	After feeding ti. ab. tx.
	#28	After lunch ti. ab. tx.
	#29	After dinner ti. ab. tx.
	#30	After meals ti. ab. tx.
	#31	#21 OR #22 OR #23 OR #24 OR #25 OR #26 OR #27 OR #28 OR #29 OR #30
	#32	Lipoproteins [ch] ti. ab. tx.

	#33	Lipoprotein particle ti. ab. tx.
	#34	Particle size [ch] ti. ab. tx.
	#35	Subclass ti. ab. tx.
	#36	High density lipoprotein [ch] ti. ab. tx.
	#37	Low density lipoprotein [ch] ti. ab. tx.
	#38	LDL ti. ab. tx.
	#39	HDL ti. ab. tx.
	#40	Small dense LDL ti. ab. tx.
	#41	sdLDL ti. ab. tx.
	#42	VLDL ti. ab. tx.
	#43	Apolipoproteins [ch] ti. ab. tx.
	#44	Lipid profile* ti. ab. tx.
	#45	Hyperlipidemia [ch] ti. ab. tx.
	#46	Cholesterol [ch] ti. ab. tx.
	#47	#32 OR #33 OR #34 OR #35 OR #36 OR #37 OR #38 OR #39 OR #40 OR #41 OR #42 OR #43 OR #44 OR #45 OR #46
	#48	#31 AND #47
	#49	Random* controlled trial ti. ab. tx. pt.
	#50	RCT ti. ab. tx. pt.
	#51	Controlled clinical trial ti. ab. tx. pt.
	#52	CCT ti. ab. tx. pt.
	#53	Random* ti. ab. tx.
	#54	Trial* ti. ab. tx.
	#55	Placebo* ti. ab. tx.
	#56	Group* ti. ab. tx.
	#57	#49 OR #50 OR #51 OR #52 OR #53 OR #54 OR #55 OR #56
	#58	#20 AND #48 AND #57
	#59	Human [ch] ti. ab. su.
	#60	Animal [ch] ti. ab. su.
	#61	#58 AND #59 NOT #60

Database	S#	Search Term
PubMed	#1	Diet, carbohydrate-restrict* [mm] ti/ab. tw.
	#2	Low carb* ti/ab. tw.
	#3	Diet, ketogenic [mm] ti/ab. tw.
	#4	Atkins ti/ab. tw.
	#5	High fat ti/ab. tw.
	#6	Keto* ti/ab. tw.
	#7	LCHF ti/ab. tw.
	#8	#1 OR #2 OR #3 OR #4 OR #5 OR #6 OR #7
	#9	Endothelium [mm] ti/ab. tw.
	#10	Vascular endothelium [mm] ti/ab. tw.
	#11	Endothelial dysfunction ti/ab. tw.
	#12	Inflammation [mm] ti/ab. tw.
	#13	Oxidative stress [mm] ti/ab. tw.
	#14	Atherosclerosis [mm] ti/ab. tw.
	#15	Coronary disease* [mm] ti/ab. tw.
	#16	Cardiovascular disease* [mm] ti/ab. tw.
	#17	Vasoconstriction [mm] ti/ab. tw.
	#18	ASCVD ti/ab. tw.
	#19	#9 OR #10 OR #11 OR #12 OR #13 OR #14 OR #15 OR #16 OR #17 OR #18
	#20	#8 AND #19
	#21	Postprandial ti/ab. tw.
	#22	Post-prandial ti/ab. tw.
	#23	Postprandial period [mm] ti/ab. tw.
	#24	Postmeal ti/ab. tw.
	#25	Post-meal ti/ab. tw.
	#26	Post-eating ti/ab. tw.
	#27	After feeding ti/ab. tw.
	#28	After lunch ti/ab. tw.
	#29	After dinner ti/ab. tw.
	#30	After meals ti/ab. tw.
	#31	#21 OR #22 OR #23 OR #24 OR #25 OR #26 OR #27 OR #28 OR #29 OR #30
	#32	Lipoproteins [mm] ti/ab. tw.

	#33	Lipoprotein particle ti/ab. tw.
	#34	Particle size [mm] ti/ab. tw.
	#35	Subclass ti/ab. tw.
	#36	High density lipoprotein [mm] ti/ab. tw.
	#37	Low density lipoprotein [mm] ti/ab. tw.
	#38	LDL ti/ab. tw.
	#39	HDL ti/ab. tw.
	#40	Small dense LDL ti/ab. tw.
	#41	sdLDL ti/ab. tw.
	#42	VLDL ti/ab. tw.
	#43	Apolipoproteins [mm] ti/ab. tw.
	#44	Lipid profile* ti/ab. tw.
	#45	Hyperlipidemia [mm] ti/ab. tw.
	#46	Cholesterol [mm] ti/ab. tw.
	#47	#32 OR #33 OR #34 OR #35 OR #36 OR #37 OR #38 OR #39 OR #40 OR #41 OR #42 OR #43 OR #44 OR #45 OR #46
	#48	#31 AND #47
	#49	Random* controlled trial ti/ab. tw. pt.
	#50	RCT ti/ab. tw. pt.
	#51	Controlled clinical trial ti/ab. tw. pt.
	#52	CCT ti/ab. tw. pt.
	#53	Random* ti/ab. tw.
	#54	Trial* ti/ab. tw.
	#55	Placebo* ti/ab. tw.
	#56	Group* ti/ab. tw.
	#57	#49 OR #50 OR #51 OR #52 OR #53 OR #54 OR #55 OR #56
	#58	#20 AND #48 AND #57
	#59	Human [mm] ti/ab.
	#60	Animal [mm] ti/ab.
	#61	#58 AND #59 NOT #60

Database	S#	Search Term
Cochrane Central	#1	Diet, carbohydrate-restrict* [mm] ti/ab/kw. tx.
	#2	Low carb* ti/ab/kw. tx.
	#3	Diet, ketogenic [mm] ti/ab/kw. tx.
	#4	Atkins ti/ab/kw. tx.
	#5	High fat ti/ab/kw. tx.
	#6	Keto* ti/ab/kw. tx.
	#7	LCHF ti/ab/kw. tx.
	#8	#1 OR #2 OR #3 OR #4 OR #5 OR #6 OR #7
	#9	Endothelium [mm] ti/ab/kw. tx.
	#10	Vascular endothelium [mm] ti/ab/kw. tx.
	#11	Endothelial dysfunction ti/ab/kw. tx.
	#12	Inflammation [mm] ti/ab/kw. tx.
	#13	Oxidative stress [mm] ti/ab/kw. tx.
	#14	Atherosclerosis [mm] ti/ab/kw. tx.
	#15	Coronary disease* [mm] ti/ab/kw. tx.
	#16	Cardiovascular disease* [mm] ti/ab/kw. tx.
	#17	Vasoconstriction [mm] ti/ab/kw. tx.
	#18	ASCVD ti/ab/kw. tx.
	#19	#9 OR #10 OR #11 OR #12 OR #13 OR #14 OR #15 OR #16 OR #17 OR #18
	#20	#8 AND #19
	#21	Postprandial ti/ab/kw. tx.
	#22	Post-prandial ti/ab/kw. tx.
	#23	Postprandial period [mm] ti/ab/kw. tx.
	#24	Postmeal ti/ab/kw. tx.
	#25	Post-meal ti/ab/kw. tx.
	#26	Post-eating ti/ab/kw. tx.
	#27	After feeding ti/ab/kw. tx.
	#28	After lunch ti/ab/kw. tx.
	#29	After dinner ti/ab/kw. tx.
	#30	After meals ti/ab/kw. tx.
	#31	#21 OR #22 OR #23 OR #24 OR #25 OR #26 OR #27 OR #28 OR #29 OR #30
	#32	Lipoproteins [mm] ti/ab/kw. tx.

	#33	Lipoprotein particle ti/ab/kw. tx.
	#34	Particle size [mm] ti/ab/kw. tx.
	#35	Subclass ti/ab/kw. tx.
	#36	High density lipoprotein [mm] ti/ab/kw. tx.
	#37	Low density lipoprotein [mm] ti/ab/kw. tx.
	#38	LDL ti/ab/kw. tx.
	#39	HDL ti/ab/kw. tx.
	#40	Small dense LDL ti/ab/kw. tx.
	#41	sdLDL ti/ab/kw. tx.
	#42	VLDL ti/ab/kw. tx.
	#43	Apolipoproteins [mm] ti/ab/kw. tx.
	#44	Lipid profile* ti/ab/kw. tx.
	#45	Hyperlipidemia [mm] ti/ab/kw. tx.
	#46	Cholesterol [mm] ti/ab/kw. tx.
	#47	#32 OR #33 OR #34 OR #35 OR #36 OR #37 OR #38 OR #39 OR #40 OR #41 OR #42 OR #43 OR #44 OR #45 OR #46
	#48	#31 AND #47
	#49	Random* controlled trial ti/ab/kw. tx.pt.
	#50	RCT ti/ab/kw. tx. pt.
	#51	Controlled clinical trial ti/ab/kw. tx.pt.
	#52	CCT ti/ab/kw. tx. pt.
	#53	Random* ti/ab/kw. tx.
	#54	Trial* ti/ab/kw. tx.
	#55	Placebo* ti/ab/kw. tx.
	#56	Group* ti/ab/kw. tx.
	#57	#49 OR #50 OR #51 OR #52 OR #53 OR #54 OR #55 OR #56
	#58	#20 AND #48 AND #57
	#59	Humans [mm] ti/ab/kw.
	#60	Animals [mm] ti/ab/kw.
	#61	#58 AND #59 NOT #60

Database	S#	Search Term
Web of Science	#1	Diet, carbohydrate-restrict* ts.
	#2	Low carb* ts.
	#3	Diet, ketogenic ts.
	#4	Atkins ts.
	#5	High fat ts.
	#6	Keto* ts.
	#7	LCHF ts.
	#8	#1 OR #2 OR #3 OR #4 OR #5 OR #6 OR #7
	#9	Endothelium ts.
	#10	Vascular endothelium ts.
	#11	Endothelial dysfunction ts.
	#12	Inflammation ts.
	#13	Oxidative stress ts.
	#14	Atherosclerosis ts.
	#15	Coronary disease* ts.
	#16	Cardiovascular disease* ts.
	#17	Vasoconstriction ts.
	#18	ASCVD ts.
	#19	#9 OR #10 OR #11 OR #12 OR #13 OR #14 OR #15 OR #16 OR #17 OR #18
	#20	#8 AND #19
	#21	Postprandial ts.
	#22	Post-prandial ts.
	#23	Postprandial period ts.
	#24	Postmeal ts.
	#25	Post-meal ts.
	#26	Post-eating ts.
	#27	After feeding ts.
	#28	After lunch ts.
	#29	After dinner ts.
	#30	After meals ts.
	#31	#21 OR #22 OR #23 OR #24 OR #25 OR #26 OR #27 OR #28 OR #29 OR #30
	#32	Lipoproteins ts.

	#33	Lipoprotein particle ts.
	#34	Particle size ts.
	#35	Subclass ts.
	#36	High density lipoprotein ts.
	#37	Low density lipoprotein ts.
	#38	LDL ts.
	#39	HDL ts.
	#40	Small dense LDL ts.
	#41	sdLDL ts.
	#42	VLDL ts.
	#43	Apolipoproteins ts.
	#44	Lipid profile* ts.
	#45	Hyperlipidemia ts.
	#46	Cholesterol ts.
	#47	#32 OR #33 OR #34 OR #35 OR #36 OR #37 OR #38 OR #39 OR #40 OR #41 OR #42 OR #43 OR #44 OR #45 OR #46
	#48	#31 AND #47
	#49	Random* controlled trial ts.
	#50	RCT ts.
	#51	Controlled clinical trial ts.
	#52	CCT ts.
	#53	Random* ts.
	#54	Trial* ts.
	#55	Placebo* ts.
	#56	Group* ts.
	#57	#49 OR #50 OR #51 OR #52 OR #53 OR #54 OR #55 OR #56
	#58	#20 AND #48 AND #57
	#59	Human ts.
	#60	Animal ts.
	#61	#58 AND #59 NOT #60
